# Efficacy of Vitamin-D supplementation in improving the prognosis of H-type hypertension in elderly patients

**DOI:** 10.12669/pjms.40.10.8464

**Published:** 2024-11

**Authors:** Sha-Sha Zang¹, Qing Zhao, Nuan Xiao, Sha Liu

**Affiliations:** 1 Sha-Sha Zang, Department of Geratology and Special Hospital Ward, The Affiliated Hospital of Hebei University, Hebei, China; 2 Qing Zhao, Department of Geratology and Special Hospital Ward, The Affiliated Hospital of Hebei University, Hebei, China; 3 Nuan Xiao, Department of Geratology and Special Hospital Ward, The Affiliated Hospital of Hebei University, Hebei, China; 4 Sha Liu, Department of Cardiovascular Medicine, The Affiliated Hospital of Hebei University, Hebei, China

**Keywords:** 25-hydroxyvitamin D, Homocysteine, H-Type Hypertension in Older Adults, Correlation

## Abstract

**Objective:**

To investigate the correlation between 25-hydroxyvitamin D and H-type hypertension in elderly patients, and to observe the clinical efficacy of vitamin D supplementation in those patients.

**Methods:**

This was a retrospective study. One hundred and twenty elderly hypertensive patients treated at The Affiliated Hospital of Hebei University from June 2022 to June 2023 were randomly divided into Group-A (n=60) with hypertension and elevated homocysteine (Hcy) levels (H-type hypertension), and Group-B(n=60) with hypertension and normal Hcy levels. Blood levels of 25-hydroxyvitamin D and 24 hours ambulatory blood pressure were assessed in both groups of patients upon admission, with the correlation analysis performed simultaneously. The therapeutic effects were compared between the two groups.

**Results:**

Through Pearson correlation analysis, there were negative correlations of serum 25-hydroxyvitamin D levels with 24 hours SSD, 24 hours DSD, dnSBP, and nDBP(all p<0.05). After 12 weeks of treatment, the treatment group had higher 25-hydroxyvitamin D levels and lower 24 hours SBP, 24h DBP, dSBP, dDBP, nSBP, nDBP levels than those of the control group(p<0.05). After treatment, the treatment group had lower blood Hcy, IMT, TC, TG, and LDL-C levels(p<0.05), and higher HDL-C levels(p<0.05) than those of the control group.

**Conclusion:**

Serum 25-hydroxyvitamin D levels in elderly patients with H-type hypertension have negative correlations with 24 hours SSD, 24 hours DSD, dnSBP, and nDBP. Oral vitamin D supplementation for H-type hypertensive patients exhibits significant therapeutic effects, with improvements in 24 hours ambulatory blood pressure monitoring results, blood lipid levels, IMT, and blood Hcy levels after treatment.

## INTRODUCTION

According to the “2020 National Bulletin on the Development of Utilities for Senior Citizens”, as of November 1, 2020, there were 264.02 million people aged ≥60 years old, accounting for approximately 18.70% of the population.^1^ As the population continues to age, the prevalence of hypertension is on the rise. Primary hypertension with elevated serum homocysteine (Hcy) levels, known as H-type hypertension, has an incidence of approximately 75%, and its pathogenesis remains unclear.^2^ There are an increasing number of studies on the correlation between vitamin D and hypertension in recent years, yet with inconsistent results.^3^-^6^ Therefore, further research and verification are needed for different regions, ethnicities, and populations. Accordingly, this study aims to investigate the correlation between 25-hydroxyvitamin D and H-type hypertension in elderly patients, and to observe the clinical efficacy of vitamin D supplementation in these patients, so as to provide new alternatives with high efficacy and safety for treating this disease.

## METHODS

This was a retrospective study. One hundred and twenty elderly hypertensive patients treated at The Affiliated Hospital of Hebei University from June 2022 to June 2023 were randomly divided into two groups: Group-A (n=60) with hypertension and elevated Hcy levels (H-type hypertension); and Group-B (n=60) with hypertension and normal Hcy levels. The deadline for data collection on May 2023. There was no significant difference in the comparison of general data between the two groups(p>0.05).Table-I.

**Table-I:** Comparison of general data between the two groups.

Groups	Age (years)	Gender (n)		Course of disease

		Male	Female	
Group-A (n=60)	63.16±4.16	31	29	6.84±1.06
Group-B (n=60)	63.67±6.97	33	27	6.99±1.08
*t/x^2^*	0.344	0.134		0.768
*P*	0.732	0.714		0.444

### Ethical Approval:

The study was approved by the Institutional Ethics Committee of The Affiliated Hospital of Hebei University (No.: HDFYLL-KY-2023-154; August 30, 2023), and written informed consent was obtained from all participants.

### Inclusion criteria:

- Patients aged 60 years;
- the treatment course. Patients who met relevant diagnostic criteria of “2018 Chinese Guidelines for Prevention and Treatment of Hypertension”^7^ and the 2016 “Expert Consensus on The Diagnosis and Treatment of H-Type Hypertension”.^8^
-Families are willing to taken to complete

### Exclusion criteria:

- Patients with secondary hypertension diseases such as primary aldosteronism;
- Patients with malignancies;
- Patients with primary organic lesions of the heart, brain, kidneys, etc.;
- Patients with mental disorders;
- Patients with abnormal comprehension;
- Patients or their families who did not agree to continue participating in the study;
- Patients with thyroid-related functional abnormalities;
- Patients with missing clinical data.

All patients were not allowed to consume food containing high animal fats and proteins within three days before blood collection. Blood samples were collected the next morning for detecting 25-hydroxyvitamin D; 24 hours systolic blood pressure (SBP), 24 hours diastolic blood pressure (DBP), daytime SBP (dSBP) and nighttime SBP (nSBP), as well as daytime DBP (dDBP)and nighttime DBP (nDBP).

Elderly patients with H-type hypertension were randomly divided into a control group and a treatment group, with 30 cases in each group. Patients in the control group were given antihypertensive drugs and oral folic acid supplementation (5mg once daily) for treatment. These patients were provided with conventional treatment according to the “Chinese Guidelines for Prevention and Treatment of Hypertension” (2018 edition), including conventional antihypertensive drugs (nifedipine controlled-release tablets, 30 mg once daily) and good healthy diet education. While patients in the treatment group received additional vitamin D (vitamin D2 softgel capsules, 800 IU, qd) based on the treatment in the control group. The treatment lasted for 12 weeks. After 12 weeks, blood Hcy, 25-hydroxyvitamin D, blood lipids, 24 hours ambulatory blood pressure, and carotid ultrasound were measured again in the enrolled patients for data comparisons and statistical analyses.

Comparisons were made between Group-A and Group-B for serum 25-hydroxyvitamin D, 24 hours SBP, 24 hours DBP, dSBP, dDBP, nSBP, and nDBP. Further comparisons were conducted between the treatment group and the control group after 12 weeks for blood Hcy, serum 25-hydroxyvitamin D, 24 hours SBP, 24 hours DBP, dSBP) dDBP, nSBP, nDBP, blood lipid indicators (TC, TG, HDL-C and LDL-C), and carotid intima-media thickness(IMT) measured by carotid ultrasound. The average of three measurements was taken as the values of the target indicators to compare the treatment efficacy. The criteria for such comparisons draw reference from the study by Zhang L et al.^9^ Both groups were followed-up for six months.

### Statistical analysis:

Software SPSS 22.0.0 was used for statistical analysis. Count data were expressed as percentages (%) and analyzed using the chi-square test (χ2).Two independent sample t-test was used for comparison between groups, and paired t-test was used to analyze data within groups. Measurement data were expressed as mean ± standard deviation (χ̅±*S*) and analyzed using the t-test. P-value < 0.05 was considered statistically significant.

## RESULTS

Group-B had higher serum 25-hydroxyvitamin D levels than Group-A (*p<* 0.05). There was no significant difference in the levels of 24 hours SBP, 24 hours DBP, dSBP, dDBP, nSBP, and nDBP between the two groups (*p>* 0.05, Table-II).Analysis of the Relationship between Serum 25-Hydroxyvitamin D and Blood Pressure. Pearson’s correlation analysis revealed a negative correlation between serum 25-hydroxyvitamin D levels and 24 hours SSD, 24 hDSD, dnSBP, and nDBP (r = -1.841, -1.762, -1.661, -1.553, -1.337, all *p<* 0.05). The correlations with H-type hypertension (Group-A) and non H-type hypertension (Group-B) were discussed separately.

**Table-II:** Comparison of serum 25-hydroxyvitamin D and blood pressurerelated indicators between Group-A and Group-B (mmHg,χ̅±*S*).

Groups	Serum 25-hydroxyvitamin D(ng/mL)	24 hours SBP	24 hours DBP	dSBP	dDBP	nSBP	nDBP
Group-A (n=60)	20.00±2.00	151.45±12.64	98.94±8.94	144.26±8.49	85.19±7.91	141.87±11.26	91.81±6.03
Group-B (n=60)	23.00±5.00	150.48±13.55	96.94±9.82	146.58±9.84	86.88±8.73	143.46±13.39	93.61±5.77
*t*	4.315	0.405	1.167	1.383	1.111	0.704	1.671
*P*	0.000	0.686	0.246	0.169	0.269	0.483	0.097

The treatment group had higher 25-hydroxyvitamin D levels than the control group (*p<* 0.05). While the former group had lower levels of 24 hours SBP, 24 hours DBP, dSBP, dDBP, nSBP, and nDBP than the latter group (*p<* 0.05, Table-III). After treatment, the treatment group had lower blood Hcy, IMT, TC, TG and LDL-C levels, but higher HDL-C levels than the control group (all p< 0.05, Table-IV).

**Table-III:** Comparison of serum 25-hydroxyvitamin D and blood pressure-related indicators between the treatment Group-And control Group-After 12 weeks of treatment (mmHg,χ̅±*S*).

Groups	Serum 25-hydroxyvitamin D (ng/mL)	24 hours SBP	24 hours DBP	dSBP	dDBP	nSBP	nDBP
Control group (n=30)	25.00±1.00	136.49±10.68	90.16±7.62	133.48±7.94	90.11±5.73	135.84±13.26	80.84±5.41
Treatment group (n=30)	49.00±1.00	131.26±10.27	85.11±6.48	128.50±6.22	86.30±3.43	128.26±10.27	77.22±6.22
*t*	92.952	0.663	2.765	2.704	3.125	2.476	2.405
*P*	0.000	0.010	0.008	0.009	0.003	0.016	0.019

**Table-IV:** Comparison of blood lipid indicators, IMT, and homocysteine levels between the treatment Group-And control Group-After 12 weeks of treatment (mmol/L,χ̅±*S*).

Groups	Blood homocysteine (µmol/L)	IMT (mm)	TC	TG	HDL-C	LDL-C
Control group (n=30)	13.14±2.04	1.34±0.34	5.18±0.77	1.69±0.51	1.52±0.33	2.95±0.67
Treatment group (n=30)	11.03±1.03	1.02±0.27	4.27±0.65	1.35±0.53	1.59±0.41	2.60±0.49
*t*	5.057	4.037	4.946	2.532	0.728	2.310
*P*	0.000	0.000	0.000	0.014	0.469	0.025

## DISCUSSION

In this study, Pearson’s correlation analysis found negative correlations of serum 25-hydroxyvitamin D levels with 24 hours SSD, 24 hours DSD, dnSBP, and nDBP (p<0.05). 25-Hydroxyvitamin D can inhibit the activity of the renin-angiotensin-aldosterone system to suppress the expression of related genes, thereby lowering the activity of the renin-angiotensin-aldosterone system and the synthesis of renin and angiotensin. Vitamin D deficiency can also lead to secondary hyperparathyroidism, which may affect internal calcium and phosphorus metabolism, leading to vascular damage and eventually causing arteriosclerosis.^10^,^11^ In patients with H-type hypertension, the high Hcy levels in the blood can aggravate small artery atherosclerosis and cause damage to the microvascular endothelium in the brain and kidneys. Further analysis showed that there are several reasons for H-type hypertension causing arteriosclerosis:

It inhibits DNA synthesis in endothelial cells, and promotes the generation of oxygen free radicals, causing damage to vascular endothelial cells, leading to a decrease in NO secretion or abnormalities in metabolic function, and reducing blood vessel elasticity and contractile capacity.

It modifies low-density lipoprotein cholesterol (LDL-C) and promotes the formation of atherosclerotic plaques through a series of reactions, such as the phagocytosis and deposition of phagocytic cells, as well as the division and repair of damaged smooth muscle cells.

It selectively inhibits the expression of thrombomodulin, enhances the activity of coagulation factors, and reduces fibrinolytic activity and antithrombin, thereby promoting platelet aggregation and leading to thrombus formation.

H-type hypertension is a common subtype of hypertension.^12^ Its prevalence has been on the rise in recent years, and this can be attributed to several factors, including improvements in living standards, changes in dietary habits and lifestyle, and an overall aging population. An increasing number of studies have shown that vitamin D can lower blood pressure by inhibiting the renin-angiotensin-aldosterone system, and preventing secondary hyperthyroidism simultaneously, providing anti-inflammatory benefits and protecting blood vessels.^13^-^15^

Research has shown that in patients with H-type hypertension, Hcy levels are closely related to SBP, DBP, and pulse pressure, and arterial pressure shows an intimate association with the occurrence, development, and prognosis of metabolic diseases.^16^ It can be explained by the relationship between serum Hcy levels and blood pressure levels. In other word, the risk of this disease may be decreased significantly if serum Hcy decreases, which is similar to the findings of this study.

Relevant studies have suggested that vitamin D deficiency and high Hcy levels are important causes of H-type hypertension and target organ damage, both of which are related to genetics and metabolism.^17^ Folic acid can reduce Hcy levels and decrease the risk of the first stroke by 18%. Supplementing vitamin D to alleviate the deficiency of 25-hydroxyvitamin D can reduce the incidence of H-type hypertension. In this study, the 24 hours SBP, 24 hours DBP, dSBP, dDBP, nSBP, and nDBP levels, as well as Hcy, IMT, TC, TG, and LDL-C levels were lower in the treatment group than those in the control group (p< 0.05); while the 25-hydroxyvitamin D and HDL-C levels were higher in the former group than those in the latter group (p< 0.05). It is believed that 25-hydroxyvitamin D is closely related to changes in blood pressure, and 25-hydroxyvitamin D is negatively correlated with increased blood pressure. As for the possible reason, 25-hydroxyvitamin D can downregulate the expression of adhesion molecules and intercellular adhesion molecule-1 in platelets and endothelial cells, downregulate the transcription of lipopolysaccharide-induced advanced glycation end product receptors and IL-6, and downregulate NF-κB and p38MAPK signaling pathways to exert anti-inflammatory effects. It is known that elevated blood pressure is closely related to plaque formation, and 25-hydroxyvitamin D can inhibit the acetylation and oxidation of LDL in macrophages, reduce foam cell formation, suppress the expression of endoplasmic reticulum-downregulated scavenger receptor A1, decrease intracellular cholesterol deposition, and exert an anti-plaque formation effect.^18^ In addition, vascular smooth muscle cells and endothelial cells both express vitamin D receptors, and vitamin D has a good inhibitory effect on arteriosclerosis. Vitamin D can regulate gene expression during the transcription process and repair the biological effects of its signaling system, thereby achieving an anti-arteriosclerosis effect.^19^. In the case of a vitamin D deficiency, it can induce a decline in endothelial function, promoting inflammatory responses, causing arteriosclerosis, increasing vascular stiffness, decreasing elasticity, and ultimately leading to changes in blood pressure.^20^

### Limitations:

The number of subjects included in this study was limited, so the conclusions drawn may not be very convincing. In addition, we only analyzed and discussed the cases included in our hospital, which may not be representative enough. We look forward to a multi-center study in the future to reach more comprehensive conclusions.

## CONCLUSIONS

Serum 25-hydroxyvitamin D levels are negatively correlated with 24-h SSD, 24-h DSD, dnSBP, and nDBP. For patients with H-type hypertension, oral supplementation of vitamin D can improve the 24 hours ambulatory blood pressure monitoring results, blood lipid levels, IMT, and Hcy levels.

### Authors’ Contributions:

**SSZ and** **QZ** carried out the studies, data collection, drafted the manuscript, and are responsible and accountable for the accuracy or integrity of the work.

**NX** performed the statistical analysis, participated in its design and did Review.

**SL** participated in acquisition, analysis, or interpretation of data and draft of the manuscript.

All authors have read and approved the final manuscript.
